# Discovery of a new class of orthosteric antagonists with nanomolar potency at extrasynaptic GABA_A_ receptors

**DOI:** 10.1038/s41598-020-66821-0

**Published:** 2020-06-22

**Authors:** Christina Birkedahl Falk-Petersen, Tsonko M. Tsonkov, Malene Sofie Nielsen, Kasper Harpsøe, Christoffer Bundgaard, Bente Frølund, Uffe Kristiansen, David E. Gloriam, Petrine Wellendorph

**Affiliations:** 1 Department of Drug Design and Pharmacology, Faculty of Health and Medical Sciences, University of Copenhagen, Universitetsparken 2, 2100 Copenhagen Ø, Denmark; 2 Translational DMPK, H. Lundbeck A/S, Ottiliavej 9, 2500 Valby, Denmark

**Keywords:** Receptor pharmacology, Ion channels in the nervous system, Drug screening

## Abstract

Brain GABA_Α_ receptors are ionotropic receptors belonging to the class of Cys-loop receptors and are important drug targets for the treatment of anxiety and sleep disorders. By screening a compound library (2,112 compounds) at recombinant human α_4_β_1_δ GABA_Α_ receptors heterologously expressed in a HEK cell line, we identified a scaffold of spirocyclic compounds with nanomolar antagonist activity at GABA_Α_ receptors. The initial screening hit **2027** (IC_50_ of 1.03 μM) was used for analogue search resulting in **018** (IC_50_ of 0.088 μM). **018** was most potent at α_3,4,5_-subunit containing receptors, thus showing preference for forebrain-expressed extrasynaptic receptors. Schild analysis of **018** at recombinant human α_4_β_1_δ receptors and displacement of [^3^H]muscimol binding in rat cortical homogenate independently confirmed a competitive profile. The antagonist profile of **018** was further validated by whole-cell patch-clamp electrophysiology, where kinetic studies revealed a slow dissociation rate and a shallow hill slope was observed. Membrane permeability studies showed that **2027** and **018** do not cross membranes, thus making the compounds less attractive for studying central GABA_Α_ receptors effects, but conversely more attractive as tool compounds in relation to emerging peripheral GABA_Α_ receptor-mediated effects of GABA e.g. in the immune system.

## Introduction

GABA_Α_ receptors (GABA_Α_Rs) are ligand-gated chloride channels belonging to the Cys-loop receptor family responsible for mediating the majority of inhibition in the CNS. The receptors are assembled from 19 different subunits (α1-6, β1-3, γ1-3, ρ1-3, ε, π and θ)^1^ with distinct combinations depending on regional brain distribution, cell type and subcellular localization^2^. The predominant GABA_Α_R subtype in the forebrain is α_1_β_2_γ_2_ which is found at synaptic locations mediating fast synaptic inhibition^2^. This is in contrast to receptors containing the δ-subunit which are located at extrasynaptic sites and mediate tonic inhibition^3^. In forebrain regions such as thalamus and hippocampus, the δ-subunit predominantly partners with the α_4_-subunit, whereas in cerebellum it exclusively partners with the α_6_-subunit^4,5^, in all cases forming receptors primarily found at extrasynaptic sites. α_5_βγ receptors are also found at extrasynaptic sites^6^ and, similar to the δ-containing receptors, they generally show a high affinity for GABA (activated by GABA spill-over from the synapse) and slow desensitization compared to synaptic receptors^3,7^.

GABA_Α_Rs are well established drug targets, with a number of approved drugs including the anxiolytic and sleep-inducing benzodiazepines, e.g. diazepam, and anaesthetics, e.g. propofol. Over the last decades, extrasynaptic GABA_Α_Rs have been the focus of many studies as they are proposed to be involved in a number of neurological disorders including epilepsy, sleep disorders, depression and stroke where changes in the tonic inhibition seem to be part of the pathology^8–11^. α_4_βδ receptors have also been found to be important for synaptic pruning as the expression of these receptors are increased in hippocampal CA1 neurons during puberty^12^. An increase in the expression of αβδ receptors, and hence higher levels of tonic inhibition in hippocampal CA1, has been linked to impaired synaptic plasticity, thus indicating that these receptors also play an important role under normal physiological conditions^13^. The α_5_βγ_2_ receptor subtype has also been found to be important for cognition and learning^14^. Altogether this posits compounds that selectively target extrasynaptic GABA_Α_Rs as interesting tool compounds and potential drug candidates.

Nonetheless, only a limited number of extrasynaptic-preferring compounds have been identified. The most renowned compound is the orthosteric agonist gaboxadol/THIP, a super-agonist at δ-containing receptors^15^, which was in clinical trials for the treatment of primary insomnia, but failed^16^. Potentially, gaboxadol is having a revival as it is currently in clinical trials for the rare disorder Angelman syndrome (trial number NCT02996305) and Fragile X (trial number NCT03697161) (as Ov101)^17^, underscoring the continued interest in targeting δ-containing receptors and tonic inhibitory current levels. In addition to the δ-preferring agonist, a number of positive allosteric modulators (PAMs) have been reported. These include delta selective compound 2 (DS2)^18^, AA-29504^19^, the flavonoid 2’MeO6MF^20,21^, and neurosteroid analogues^22,23^, however none of these discriminate between α_4_βδ and α_6_βδ receptors. Interestingly, no truly selective antagonists for δ-containing receptors have been identified although the competitive antagonist DPP-4-PIOL was found to be approximately 20 times more potent in inhibiting tonic over phasic currents (IC_50_ of 0.87 nM) in rat slice recordings from dentate gyrus granule cells^24^. As a whole, inhibitors of extrasynaptic GABA_Α_Rs would constitute highly useful pharmacological tool compounds, and potentially be of therapeutic relevance in e.g. functional recovery after stroke and certain types of absence epilepsies^10,25^.

Here we used our recently implemented cell-based assay, measuring changes in membrane potential by fluorescence^26^, to search for novel α_4_βδ receptor antagonists using α_4_β_1_δ GABA_Α_Rs as a model receptor to screen an in-house assembled small-molecule compound library, and report the identification of a novel class of antagonists with a clear competitive profile and preference for α_3,4,5_-containing extrasynaptic GABA_Α_Rs with low to mid nanomolar potency.

## Results

### Validation of FMP assay for compound screening

We have previously established a FLIPR membrane potential (FMP) assay to study recombinant δ-containing GABA_Α_Rs entailing a HEK293 Flp-In cell line stably expressing the human δ-subunit  which is suitable for co-transfection with any desired α/β subunits^26^. Initially, we validated the applicability of the FMP assay for efficient compound screening in 96-well microtiter plates using the human α_4_β_1_δ GABA_Α_R heterologously expressed in HEK cells. This receptor was chosen as we have previously shown this system to be reliable in expressing δ-containing receptors^26^. To this end, we determined the Z′-factor, which is a statistical factor used to measure the suitability of an assay for high throughput screening^27^. An assay is regarded an excellent assay when the value of the Z′-factor is in the range of 0.5 ≤ Z′ < 1, as this indicates a large separation between positive and negative controls^27^. The Z′-factor was determined to 0.68 (three independent experiments with 48 positive and negative control wells distributed evenly across the ligand plate), thus categorizing the FMP assay on α_4_β_1_δ receptors as an excellent assay for screening with high sensitivity and reproducibility.

### Primary screening using the FMP assay

**Figure 1:**
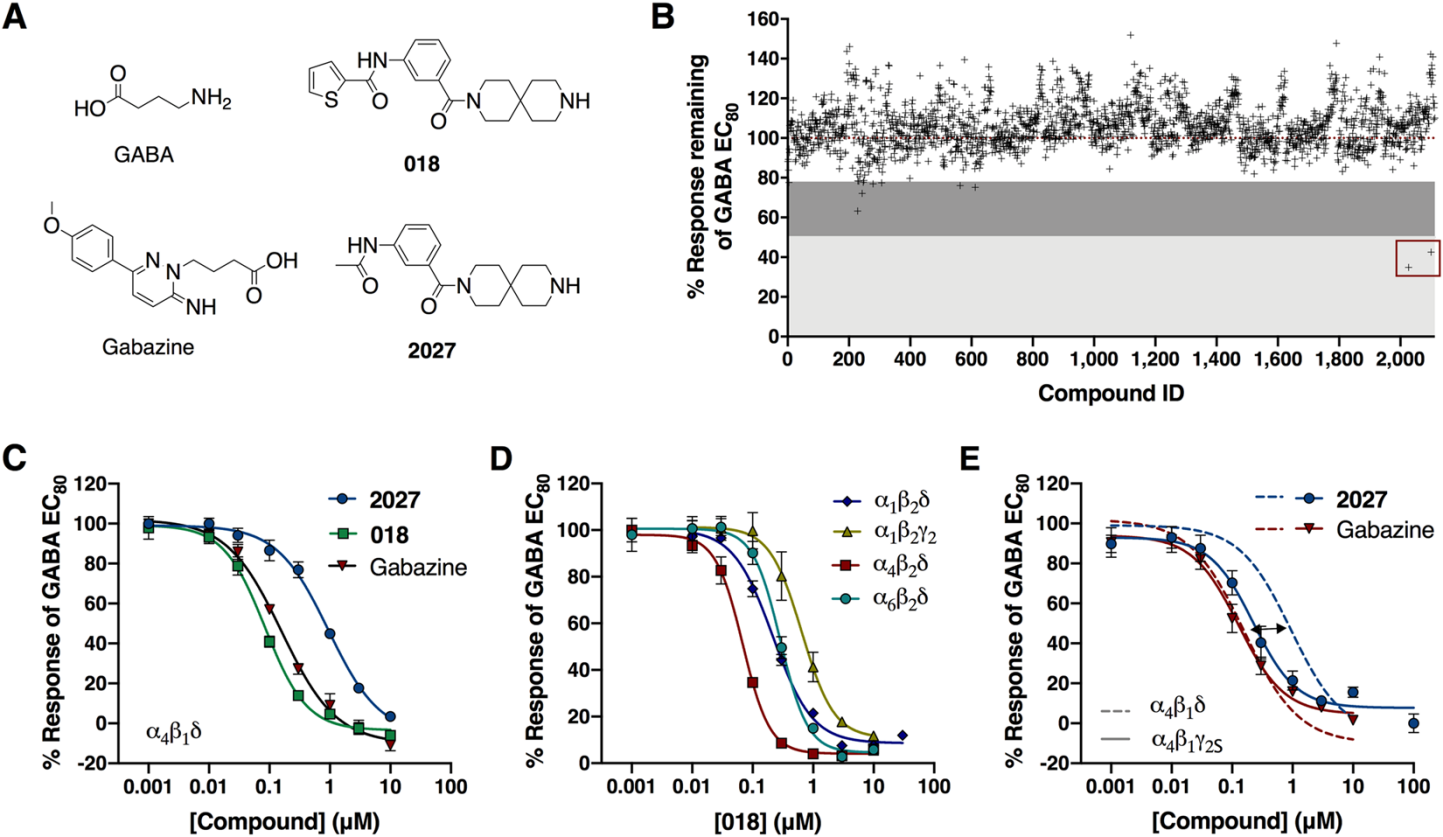
The identified small-molecule ligands **2027** and **018** are antagonists at GABA_Α_Rs. **(A)** Chemical structures of **018**, **2027**, GABA and Gabazine. **(B)** Results of primary screening of the compound library at α_4_β_1_δ receptors in antagonist mode in the FMP assay (96-well format). Data shown are normalized to GABA EC_80_ of compounds tested in 10 μM in singlicates. (**C)** Concentration-response curves of **2027** and **018** at α_4_β_1_δ receptors, with gabazine shown as reference. (**D)** Concentration-response curves of **018** at selected GABA_Α_ receptor subtypes showing α-subunit dependent inhibition of the GABA EC_80_ signal. (**E)** Concentration-response curves of **2027** and gabazine at α_4_β_1_δ and α_4_β_1_γ_2_ showing a subtype-dependent right shift of the curve only for **2027**. Data are shown as representative curves obtained in the FMP assay with three technical replicates (means ± SD). Collected IC_50_ values ± SEM and GABA EC_80_ values are given in Table 1.

The compound library was screened for antagonist activity at α_4_β_1_δ receptors in the FMP assay in a single concentration of 10 μM in singlicates by applying the compounds together with a concentration of GABA corresponding to GABA EC_80_ (Table 1). In the primary screening, 10 compounds inhibited the GABA EC_80_ signal by more than 22%, but only two compounds, compound **2027** (*N*-(3-(3,9-diazaspiro[5.5]undecane-3-carbonyl)phenyl)acetamide) and **2100** (1-(furan-2-ylmethyl)-*N*-(2-methoxyphenyl)piperidine-4-carboxamide), were able to inhibit the signal by more than 50% (Fig. 1A,B). In the following hit validation, the same 10 compounds were tested in concentrations of 100, 50 and 10 μM in triplicates, in which only **2027** showed antagonistic effect, prominent already at a concentration of 10 μM (See Supplementary Fig. S1). Thus, we went on to further characterize **2027** by making a full concentration-inhibition curve. **2027** was able to completely inhibit the GABA EC_80_ -induced response in a concentration-dependent manner at α_4_β_1_δ receptors with an IC_50_ value of 1.03 μM (Fig. 1C, Table 1). Gabazine was included as reference compound having an IC_50_ value of 0.24 μM (6.61 ± 0.058), (n = 3) at α_4_β_1_δ receptors (Fig. 1A,C).

**Table 1:** Antagonist activity of **2027** and **018** plus GABA agonist potencies at selected GABA_Α_ receptor subtypes determined in the FMP assay.

Receptor	2027 (IC_50_ (μM), pIC_50_ ± SEM, n)		018 (IC_50_ (μM), pIC_50_ ± SEM, n)		GABA (EC_50_ (μM), pEC_50_ ± SEM, n)		GABA EC_80_, μM
α_1_β_2_δ	6.68	(5.17 ± 0.10, 3)	0.24	(6.61 ± 0.050, 4)	6.71	(5.17 ± 0.087, 4)^a^	10–14
α_1_β_2_γ_2_	4.96	(5.30 ± 0.17, 4)	0.79	(6.10 ± 0.11, 4)	1.71	(5.77 ± 0.022, 3)	8.0
α_2_β_2_γ_2_	2.96	(5.53 ± 0.19, 3)	0.32	(6.49 ± 0.13, 3)	1.47	(5.83 ± 0.045, 3)	10
α_3_β_2_γ_2_	0.29	(6.54 ± 0.17, 3)	0.079	(7.10 ± 0.18, 3)	2.21	(5.63 ± 0.11, 3)	10
α_4_β_1_δ	1.03	(5.99 ± 0.028, 3)	0.088	(7.06 ± 0.11, 4)	0.17	(6.77 ± 0.11, 3)	0.27–1.0
α_4_β_1_γ_2_	0.17	(6.78 ± 0.081, 3)	0.037	(7.43 ± 0.094, 3)	1.40	(5.86 ± 0.014, 3)	4.0–8.0
α_4_β_2_δ	0.36	(6.44 ± 0.12, 3)	0.068	(7.17 ± 0.080, 3)	0.25	(6.61 ± 0.030, 3)^a^	0.80–1.0
α_5_β_2_γ_2_	0.59	(6.23 ± 0.19, 4)	0.051	(7.29 ± 0.19, 4)	0.47	(6.33 ± 0.20, 3)	3.0
α_6_β_2_δ	4.13	(5.38 ± 0.050, 3)	0.33	(6.48 ± 0.082, 3)	0.21	(6.68 ± 0.13, 3)^a^	3.0

The antagonist activity was determined using the stated GABA EC_80_ concentration calculated from the determined GABA potencies.

IC_50_/EC_50_ values are determined from concentration response curves and given as means. ^a^Data from L’Estrade *et al*.^49^.

### Identification of the hit analogue 018 displaying increased potency

Next, we identified 52 structural analogues of compound **2027** by substructure searches in a database of commercially available compounds, which were tested for antagonist activity in the FMP assay in concentrations of 5 μM and 0.5 μM (For structures and data see Supplementary Figs. S2 and S3). Of the 52 analogues, 20 showed antagonist activity at 5 μM, and of these, 14 were found to be equipotent or more potent than compound **2027**. Of the 20 active analogues, **018 (***N*-(3-(3,9-diazaspiro[5.5]undecane-3-carbonyl)phenyl)thiophene-2-carboxamide) was identified as the most potent compound with an IC_50_ value of 0.09 μM at α_4_β_1_δ, thus being approximately 10 times more potent than **2027** (Fig. 1C, Table 1). Interestingly, all identified active compounds were found to have a common 3,9-diazaspiro[5.5]undecane moiety (Fig. 1A). Thus, based on this common structural motif, additional 44 analogues were purchased, but none of these showed to be more potent than **018** (Data not shown). We thus decided to use **018** alongside **2027** as model compounds to look further into the pharmacological properties of this new class of GABA_Α_R antagonists.

### Compound 018 shows preference for extrasynaptic GABA_Α_ receptor subtypes

To assess if the primary hit **2027** and the more potent analogue, **018** (Fig. 1A), displayed any subtype selectivity we wanted to determine the potency at receptor subtypes in which either α, β or γ/δ subunits were interchanged. We chose subtype combinations that were both informative and physiologically relevant. Thus α_1−3, 5_ were expressed together with the γ_2_ subunit and α_4,6_ with the δ-subunit^1^. Additionally, α_1_β_2_δ and α_4_β_1_γ_2_ receptors were included, as they are relevant for comparison and found to be expressed in hippocampus^28,29^. Further, we chose to use the β_2_-subunit for most receptors as it is convenient to express and because expression of β_3_ leads to homomers in our cell system^26^ (Table 1). First, we determined the GABA EC_50_ values at all the selected receptors in order to calculate the concentrations corresponding to GABA EC_80_. These values are stated in Table 1, and are overall similar to values determined by others at human subtypes expressed in HEK cells^30^, with GABA being more potent at the extrasynaptic and δ-containing receptors compared to γ-containing synaptic receptors. Next, we determined and compared the potencies of **2027** and **018** at the selected receptors. From these comparisons, we found that the observed potency depends most strongly on the specific α-subunit (Fig. 1D, Table 1). **018** was most potent at α_3−5_ containing receptors, with potencies in the mid nanomolar range (37–88 nM) whereas the potency at α_1,2,6_ receptors were in the high nanomolar range (240–790 nM). From this it follows that at a concentration of 10–100 nM, **018** would result in more than 50% inhibition of the α_3–5_ containing receptors but less than 20% inhibition at the α_1,2,6_ containing receptors. Comparing the potency at α_4_β_2_δ receptors (IC_50_ of 68 nM) and α_1_β_2_γ_2_ receptors (IC_50_ of 790 nM) showed 12 times increased potency of **018** at the α_4_- compared to the α_1_-containing receptors. The potency ranking based on α-subunit, α_4_ = α_5_ = α_3_ > α_2_ > α_6_ = α_1_, indicates a preference for the forebrain extrasynaptic GABA_Α_Rs, often carrying α_4_ (Fig. 1D, Table 1). Overall, a similar trend in potency order was seen for **2027** albeit right-shifted 5–10 times, except for α_1_β_2_δ, where the potency was 28 times reduced for **2027** compared to **018** (Table 1). Finally, to assure that the observed subtype differences were not coincidental, we compared with data from the non-subtype selective antagonist, gabazine, tested at α_4_β_1_γ_2_ cf. α_4_b_1_δ subtypes. As shown in Fig. 1E, gabazine has a similar IC_50_ value at both subtypes (IC_50_ of 0.11 μM (6.94 ± 0.021), n = 4 and 0.24 μM, respectively), whereas **2027**, by comparison, has a clear preference for α_4_β_1_δ.

### Compound **018** is a competitive antagonist

**Figure 2:**
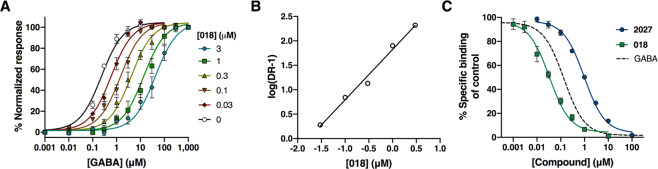
Compound **018** is a competitive antagonist at α_4_β_1_δ receptors and displaces [^3^H]muscimol binding. (**A)** Schild plot of **018** at α_4_β_1_δ receptors determined in the FMP assay showing a right shift of the GABA concentration-response curve with increasing concentrations of **018** without affecting efficacy. Shown are pooled data from five independent experiments with three technical replicates given as means ± SEM. (**B)** Schild plot of data displayed in (**A)** showing a Schild slope of 1.03 indicative of competitive antagonism. (**C)** Concentration-dependent inhibition of [^3^H]muscimol binding by **018** and **2027** in rat cortical homogenate, with GABA as reference, showing a competitive profile of the compounds. Data are given as means ± SEM from four independent experiments performed in technical triplicates. The K_i_ of GABA is 0.049 μM, data from Krall *et al*.^48^.

To determine the type of antagonism displayed by **018**, we performed a Gaddum/Schild analysis at α_4_β_1_δ receptors in the FMP assay. Thus, GABA concentration-response curves were generated in the absence or presence of increasing concentrations of **018** ranging from 0.03 μM to 3 μM. This resulted in a decrease in the potency of GABA with increasing concentrations of **018** (right-shift of curves) but without any changes in the slope and/or the efficacy of GABA at any of the tested concentrations of **018** (Fig. 2A). The Schild slope for **018** was determined to 1.03 [0.96–1.06], (n = 5) and was not significantly different from unity (P = 0.668), indicating that **018** is a competitive antagonist at α_4_β_1_δ receptors (Fig. 2B). Additionally, the K_Β_ for **018** was determined to 19 nM [0.0138; 0.0278] from the Schild analysis. To further support the finding that **018** is a competitive antagonist, its ability to inhibit radioligand binding of [^3^H]muscimol to rat brain cortical homogenate was tested by performing competition binding studies. These experiments showed that **018** and **2027** were able to fully inhibit the binding of [^3^H]muscimol with K_i_ values of 20 nM (7.71 ± 0.06), (n = 4) and 0.56 μM (6.25 ± 0.06), (n = 4), respectively (Fig. 2C). The determined K_Β_ and K_i_ values correspond very well supporting a competitive profile of the compounds.

### Validation using whole-cell patch-clamp electrophysiology

**Figure 3:**
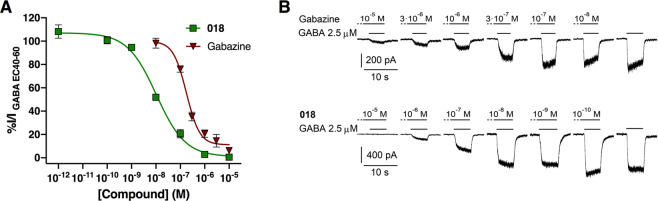
Whole-cell patch-clamp electrophysiology studies of α_4_β_1_δ receptors support the antagonism determined in the FMP assay. (**A) 018** concentration-dependently inhibits the current induced by 2.5 μM GABA (corresponding to GABA EC_30-40_) at α_4_β_1_δ receptors with an IC_50_ of 10.8 nM and a Hill slope of −0.74. Gabazine was included as reference with an IC_50_ of 0.18 μM and a Hill slope of −1.53. (**B)** Representative current traces for **018** and gabazine. The dotted line represents 30 sec pre-application of the antagonist. Data are shown as pooled data from 5–7 cells given as means ± SEM and normalized to the GABA 2.5 μM control.

To validate the results from the FMP assay, compound **018** was tested in whole-cell patch-clamp electrophysiology at α_4_β_1_δ receptors conveniently expressed in the same δ-HEK cell line as used for the FMP assay. Again, **018** was found to be a full antagonist and the IC_50_ value was determined to 10.8 nM [6.27; 14.7], (n = 7), which is 9 times more potent than determined in the FMP assay. The Hill slope for **018** was found to be −0.74 [−0.95; −0.54] (Fig. 3), which is more shallow than what would be expected for a competitive GABA_Α_R antagonist^31^. To test if the shallow Hill slope was an effect of the compound or the assay, we tested the classical competitive antagonist gabazine using the same setup. The IC_50_ of gabazine was determined to 0.18 μM [0.11; 0.29], (n = 5) with a Hill slope of −1.54 [−2.2; −0.84] (Fig. 3). This is significantly steeper than that of **018** (**P = 0.0099, unpaired t-test), thus indicating that the shallow Hill slope of **018** is not a technical issue but an effect of the compound.

### Compound **018** displays slow dissociation kinetics

**Figure 4:**
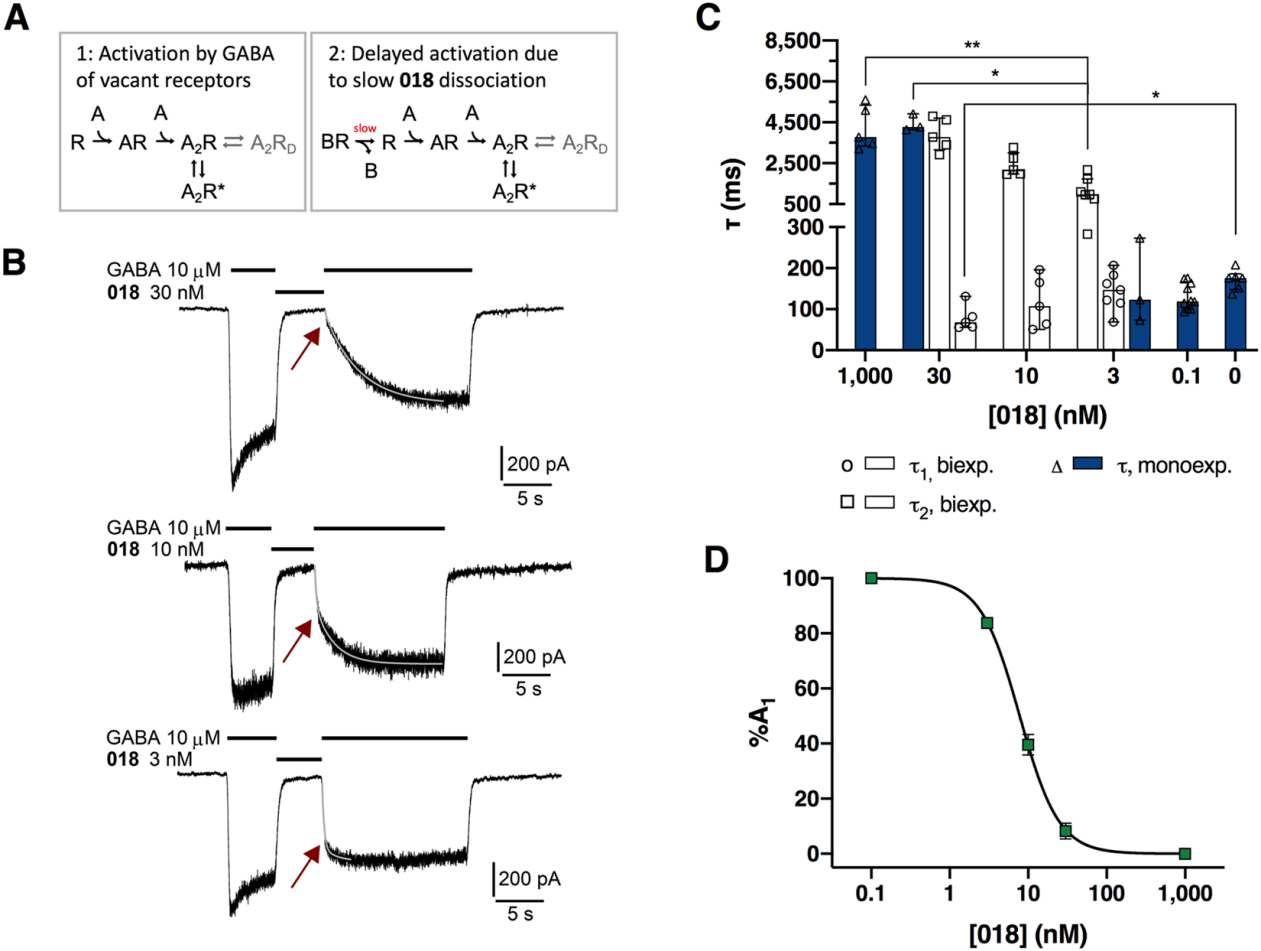
Compound **018** displays slow dissociation kinetics. (**A)** Simple kinetic models for the activation by GABA of vacant receptors (Model 1) and receptors initially blocked by **018** (Model 2), respectively. When present, dissociation of **018** is the slowest (rate-limiting) step and delays the overall process of activation by GABA. Openings are assumed to occur only with two agonist molecules bound (A_2_R* state) and desensitization (A_2_R_D_, in grey) to be of minor importance during the activation phase. (**B**) Examples of current traces for 2.5 μM GABA before and after 5 sec application of 10 nM **018** showing slow dissociation kinetics of **018**. A biexponential function (grey line) was fitted to the activation phase of the current traces, with the fast component interpreted as reflecting GABA activation of vacant receptors (Model 1), and the slow component reflecting receptors, where activation had to await dissociation of **018** (Model 2). The arrow indicates the break point where the fast component is essentially completed. (**C)** Time constants, τ, for currents induced by 2.5 μM with or without pre-application of **018**. Constants determined by fitting to a monoexponential function are shown as blue bars (Δ) and biexponential fittings in white bars (**☐**,**○**). Data are shown as median ± interquartile range for 5–10 cells. Statistical analysis was performed using Kruskal-Wallis ANOVA follow by Dunn’s multiple comparison. τ_1_ is compared to 0 (2.5 μM GABA without pre-application of **018**) and for τ_2_ all values are compared. (**D)** The contribution from the fast-rising amplitude %A_1_ concentration dependently decrease for increasing concentrations of **018** using 2.5 μM GABA, giving a functional K_B_ of 7.80 nM. %A_1_ was determined from the fitting a to biexponential function using data displayed in (**C**). The fractional amplitude of the fast component (%A_1_) is a measure of the fraction of receptors that are vacant when GABA is applied. For values see Table 2. Additional data for similar experiment using 100 μM GABA is given in supplementary Fig. S4 and Table S1.

From the patch-clamp current traces of the concentration-response curve of **018** it was apparent that the presence of **018** concentration-dependently delayed the activation phase of GABA-activated currents (Fig. 3B). For concentrations of **018** close to its IC_50_ value, it was further apparent that the activation phase followed a biphasic time course. In order to obtain detailed information on the dissociation kinetics of **018** we decided to further investigate the time-course of GABA activation in the presence of **018**. To this end, we tested concentrations of **018** ranging from 0.1 to 1000 nM pre-applied before application of 2.5 μM GABA, and determined the number of exponential components with their time constants and amplitudes that best fitted the time course of the activation phase (Fig. 4B+C, Table 2). The results show that the time course of the activation phase of the GABA currents could be described well using mono- or biexponential functions. Thus, it confirmed our initial observations, as the activation phase of the GABA current after preapplication of 3, 10 and 30 nM **018** could be fitted using biexponential functions, except for 6 of the tested cells that were fitted by mono-exponential functions (Fig. 4C, Table 2). At a concentration of 30 nM, four out of five tested cells could be fitted to both a mono- and a biexponential function, but statistical analysis showed that all were fitted best by the biexponential function (*F*-test, P < 0.001). The GABA activation phases with 0.1 nM and 1000 nM **018** could only be fitted using mono-exponential functions, as the slow and fast component, respectively, were virtually absent at these concentrations. The determined activation time constants are given in Table 2 and Fig. 4C. The time constant for the fast component, τ_1_, for 0.1–10 nM **018** was not significantly different from the single time constant, τ, for 2.5 μM GABA alone, but for 30 nM **018**, the determined τ_1_ of 68.0 ms was significantly lower compared to GABA alone (P = 0.0068, Kruskal-Wallis ANOVA followed by Dunn’s multiple comparison). Comparing the time constants of the slow component, τ_2_, determined with the different concentrations of **018** showed that the time constant τ_2_ for 3 nM **018** was significantly lower than for 1000 nM and 30 nM (P = 0.0066 and P = 0.024 respectively, Kruskal-Wallis ANOVA followed by Dunn's multiple comparison). Additional experiments using 100 μΜ GABA showed that the determined slow time constants, τ_2_, were not significantly different from those using 2.5 μM GABA (Multiple t-test) (See Supplementary Fig. S4 and Table S1).

**Table 2:** Summary of the time constant determined for 2.5 μM GABA with and without preincubation with **018** and calculated percentage contribution of the fast-rising current (%A_1_) relative to the total induced current.

018 (nM)	τ_1_ (ms)	τ_2_ (ms)	%A_1_	n
0	174 [148;186]^a^	—	—	6
0.1	119 [101;167]^a^	—	—	10
3	147 [115;183] 123 [73.3;273]^a^	1090 [960;2180] —	83.8 —	7 3
10	107 [57.5;180]	2170 [1970;3010]	39.6	5
30	68.0 [56.3;106] —	3780 [3160;4710] 4270 [4140;4920]^a^	8.3 —	5 3
1000	—	3780 [3330;5350]^a^	—	5

If not otherwise stated, current traces were best fitted using a biexponential function. Data are given as medians followed by 25–75% quartiles in squared brackets. n denotes the number of tested cells. Statistics are shown and described in Fig. 4B. ^a^Current traces were best fitted using a monoexponential function.

For those activation phases of the GABA current best fitted using a biexponential function, we determined the fraction of the total current amplitude contributed by the fast component (%A_1_) (Fig. 4D, Table 2). For 1000 nM and 0.1 nM, which could only be fitted using mono-exponential functions (slow and fast, respectively), the %A_1_ were set to 0% and 100%, respectively. This revealed a decrease in the relative contribution from the fast component with increasing antagonist concentrations (Fig. 4D). We interpret these observations based on the models presented in Fig. 4A, which are simple kinetic models for activation of receptors by GABA, and are based on models by Mortensen et al. and Keramidas & Harrison, for α_4_β_3_δ and α_4_β_2_δ receptors^32,33^, respectively, assuming that α_4_β_1_δ receptors display similar kinetics. Model 1 describes the activation of vacant receptors by GABA and Model 2 describes the situation when receptors are initially blocked by binding of **018**. When receptors are bound to **018**, the number of vacant receptors that GABA can activate will be reduced depending on the concentration and affinity of **018**, thus delaying the overall process of activation, with the dissociation of **018** being the rate-limiting step. Assuming that the fast component of activation is due to GABA activating the population of initially vacant receptors, it follows that the decrease in the relative contribution of the fast component (%A_1_) with increasing antagonist concentrations (Fig. 4D) must reflect a decrease of the fraction of vacant receptors and a corresponding increase in fraction of receptors that are occupied by antagonist at the end of the pre-application.

Specifically, a %A_1_ of 50% should correspond to a receptor occupation by **018** of 50%. The corresponding concentration of **018** is then a measure of affinity, a “functional K_Β_“, provided that binding equilibrium is obtained during pre-application. In this way, the functional K_Β_ for **018** at α_4_β_1_δ receptors was determined to 6.90 nM [5.99; 7.91] for 2.5 μM GABA. The same experiment was performed using 100 μM GABA giving a similar result with a functional K_Β_ of 7.80 nM [6.93; 8.73] (See Supplementary Fig. S4 and Table S1). These values concur both with the determined K_Β_ from the Schild analysis and K_i_ from the binding studies.

### 018 and 2027 display very low membrane permeability

To investigate the potential of both **018** and **2027** for *in vivo* studies, the compounds were tested in an *in vitro* permeability model using MDCK-MDR1 cells, to assess the bidirectional permeability. Results from these experiments indicate very low permeability of both **018** and **2027**. The apical to the basal permeability was 0.00 cm/s for both of the compounds whereas the basal to apical permeability was 0.79 and 0.67 10^−6^ cm/s for **018** and **2027**, respectively. Additionally, based on a high recovery both before and after (above 85%), sticking of compounds to the cells is not an issue (See Supplementary Table S2)”. This indicates that the compounds most likely will not pass cell membranes and thus not get into the brain.

## Discussion

We have identified a new class of competitive GABA_Α_R antagonists with preference for α_3,4,5_-containing extrasynaptic receptors typical in forebrain regions such as cortex, thalamus and hippocampus. Having identified **2027** as a low-micromolar GABA_Α_R antagonist, we were able to increase the potency approximately 10 times by testing of analogues with the identification of **018**. This confirmed the predictions of the library, which was designed to contain compounds that had at least 50 commercially available structural analogues, with the purpose of increasing potency of the primary hit and narrowing down the searchable chemical space.

Through the testing of analogues of **2027**, we identified the 3,9-diazaspiro[5.5]undecane moiety as an important structural element for antagonist activity. Interestingly, this structure does not contain the carboxylic acid/GABA moiety like typical orthosteric GABA_Α_R antagonists like gabazine^34^ and DPP-4-PIOL^24^. It was thus interesting that the Schild analysis and binding studies of the compounds convincingly showed a competitive profile. However, there are other examples of orthosteric GABA_Α_R antagonists lacking the carboxylic acid, including bicuculline and the acid-sensing ion channel type 3 agonist 2-guanidine-4-methylquinazoline (GMQ)^35^, underlining that the presence of a “GABA”-like moiety is not necessary for binding to the orthosteric site. Additionally, we found that further substitution on the secondary amine of the 3-substituted 3,9-diazaspiro[5.5]undecane structure abolished the activity of the compounds (Supplementary Figs. S2 and S3), indicating that this part of the structure is important. By comparing the structures of **018** and **2027** it is clear that there is space for rather large substituents in the 3-position of the 3,9-diazaspiro[5.5]undecane. The studies of **018** and **2027** at selected GABA_Α_ receptor subtypes showed that the α-subunit dictates potency. Comparing the IC_50_ values we found that **018** is more potent at α_3,4,5_-subunit containing receptors than the α_1_-containing receptors, regardless of the presence of either the δ or γ subunit and the type of β-subunit. This conclusion could be strengthened with determination affinity-based K_B_ values for all subtypes. A study by Ebert et al. looked into the α-subunit selectivity of the known competitive antagonist gabazine, bicuculline and Thio-4-PIOL at αβγ_2_ receptors, but did not find any differences between the receptor subtypes when studying functional responses^36^. It is therefore interesting that this new class of GABA_Α_R antagonists seemingly discriminate between the different α-subunits. It is however known that the GABA sensitivity is dependent on the type of α-subunit^37^ which we also show here. Interestingly, there is no clear correlation between the GABA potency and those of **2027** and **018** at the different subtypes, thus underlining that the compounds might also interact with residues outside the GABA binding site that are important for the potency, e.g. via hydrophobic interactions of the distal amide and aromatic moieties assuming that the protonated 9-amino group has the same interactions as GABA and thus is located under the c-loop.

The competitive profile of **018** was corroborated by similar affinity constants obtained from the Schild analysis, radioligand binding studies, and electrophysiology, although the specific receptor subtypes present in the native membranes is not equivocal. It has previously been shown that there is good correlation between affinity and potency for GABA_Α_R antagonists^36^, as shown here for both **018** and **2027**. However, as the data can be fitted to a one-site binding model, this indicates binding to either a uniform receptor population or only high-affinity δ-containing receptors because the assay protocol employed only a low nanomolar [^3^H]muscimol concentration^38^. Immunoprecipitation studies from cortex have shown that the δ-containing receptors constitute around 10% of the total GABA_Α_Rs^39^, thus only making up a small part of the available binding sites. Thus, at this point we cannot rule out that the differences observed for the α-subunits are only functional.

Patch-clamp electrophysiology studies of **018** corroborated the antagonistic profile from the FMP assay, however with a 9 times increased potency, likely relating to assay differences. Whole-cell patch-clamp is more sensitive and gives more details compared to the FMP assay, and has the advantage of measuring the response of a single cell in contrast to an average of cells in a specific well as in the FMP assay. It has previously been reported that the results between the two assays differ^18,40^. Additionally, we have also previously reported 5–8 times in potency differences of agonists between the two assays^26^. This difference however does not seem to be consistent, as the potency determined for gabazine in this study was similar in the two assays, with IC_50_ values of 0.18 μM and 0.24 μM in whole-cell patch-clamp and FMP, respectively. In the FMP assay there is the potential of compounds interfering with the fluorescence which may skew responses. However, both **2027** and gabazine do not interfere with the fluorescence signals in concentrations below 10 μM, (Supplementary Fig. S5) ruling out this as a cause of the discrepancy. Additionally, we used a GABA EC_80_ concentration in the FMP assay compared to GABA EC_40_ in patch-clamp to avoid desensitization of the response.

From the patch-clamp experiments we observed a surprisingly shallow Hill slope for **018** compared to the classical orthosteric antagonist gabazine when using the same cells and setup. Therefore, it is more likely that the shallow Hill slope is a compound effect, which is interesting as both the Schild analysis and binding studies confirmed that the compound is competitive and thus must share or at least have an overlap with the GABA binding site. Further, it does not seem likely that the slow dissociation kinetics of the compound is the cause, as Vestergaard *et al*.^41^ did not observes differences in Hill slopes for a series of analogues (competitive antagonists) of the partial GABA_Α_R agonist 4-PIOL, which included both fast and slowly dissociating antagonists. Thus, we have not been able to find an explanation for the shallow Hill slope other than being a characteristic of the compound.

The kinetics of GABA currents in the presence of **018** were studied in order to obtain additional information on the interaction of **018** with the α_4_β_1_δ receptor. The observation, that the activation phase of GABA currents in the presence of **018** consisted of two exponential components, was interpreted as GABA interacting with two “populations” of receptors: those that are initially vacant and those that are initially occupied with antagonist, giving rise to the fast and slow, respectively, component of the activation phase (Fig. 4A,B). This is a simplification because, due to the reversibility and branching of the underlying processes (binding, gating, desensitization), the two populations do not remain distinct in time. Nevertheless, we found indications that the interpretation of the parameters obtained from biexponential fitting of the activation time course is indeed meaningful: First, the time constant of the fast component of activation, τ_1_ (interpreted as GABA activation of vacant receptors), is similar to the time constant for activation by GABA alone, both with the high and low GABA concentration used. Second, the time constant of the slow component of activation, τ_2_ (interpreted as reflecting the rate limiting antagonist dissociation), is independent of the GABA concentration and largely independent of the antagonist concentration. A trend towards faster τ_2_ with lower concentrations of **018** was observed for both 100 and 2.5 μM GABA. However, this can be explained by the fact that at the lower concentrations of antagonist, the fractional amplitude of the slow component (%A_2_) is small, thus giving less reliable estimates of the τ_2_. Third, the fractional amplitude of the fast component, %A_1_, interpreted as the fraction of receptors that are vacant and immediately available for GABA to bind to and activate, decreases with the antagonist concentration, thus reflecting the concentration-dependent binding of **018** before application of GABA. The functional K_B_ obtained from this relationship is similar with both 2.5 μM and 100 μM GABA and in agreement with the K_i_ determined from receptor binding and the K_Β_ from functional experiments (radioligand binding and FMP with Schild analysis). Also, the potential influence of drug application needs to be considered, since the solution exchange rate sets a limit to how fast processes can be resolved. With saturating GABA concentration, time constants as short as 35 ms were obtained, and therefore we conclude that solution exchange does not influence the slower τ values obtained in the kinetic experiments and analysis. This allows us to conclude that the time constant of the slow component of the GABA activation time course (τ_2_) obtained in the presence of **018** is an estimate of the dissociation time constant of **018**. Thus, **018** is characterized as slowly dissociating with a time constant of the same order of magnitude as the most slowly dissociating GABA_Α_ antagonists described^41^.

To address future *in vivo* usage of the compounds, the cellular permeability of **2027** and **018** were assessed and found to be very low, indicating that neither of these compounds will likely pass the blood-brain barrier, thus limiting their use for* in vivo* CNS studies. Nonetheless, as several studies have shown that GABA_Α_R subunits are expressed outside CNS, on for example several types of peripheral immune cells^42^ and that GABA_Α_Rs can be potential targets for cancer therapy, this is not discouraging as such. For these purposes it would be an advantage to have compounds that do not get into the brain, as this will limit unwanted neurological side-effects. Thus, a compound such as **018** would be highly interesting to examine for such peripheral GABA_Α_R-mediated inhibitory effects and may have a favourable pharmacokinetic profile because of the slow dissociation kinetics. Furthermore, it would be of interest to probe the binding site further, e.g. by the development of a radiolabelled analogue, and/or computational modelling to gain more information on the binding site residues and, ultimately, the α-subunit dependence. Lastly, the α_3,4,5_-extrasynaptic receptor preference displayed by **018** presents an alternative, and highly desired, way to study (*in vitro*) tonic inhibition in the forebrain, e.g. by slice electrophysiology. Interestingly, the functional selectivity of the identified compounds for extrasynaptic over synaptic receptors make them promising tools, especially in the absence of δ-selective antagonist.

## Methods

### Materials

Dulbecco’s modified Eagle medium (DMEM) containing GlutaMAX-I, foetal bovine serum (FBS), Dulbecco’s phosphate-buffered saline (DPBS), penicillin-streptomycin, hygromycin B, trypsin-EDTA, and Hank’s balanced salt solution (HBSS) were all purchased from Life Technologies (Paisley, UK). Polyfect transfection reagent was purchased from Qiagen (West Sussex, UK). FLIPR membrane potential Blue dye was purchased from Molecular Devices (Crawley, UK). Buffer components, poly-D-lysine (PDL) and 4-(2-hydroxyethyl)-piperazine-1-ethanesulfonic acid (HEPES) were purchased from Sigma-Aldrich (St. Louis, MO, USA). GABA and gabazine were from Tocris Bioscience (Bristol, UK). Compounds in the screening library and analogues were purchased from either Enamine, Vita-M or ChemBridge as stated in the supplementary information. [^3^H]muscimol (36.9 Ci/mmol) was purchased from PerkinElmer (Waltham, MA, USA). Compounds **2027** and **018** can be purchased from Enamine and have ID numbers Z1839922409 and Z1839935473, respectively.

### Compound design and analogue search

Starting from Enamine’s > 2 million in-stock compounds, a diverse library of 2,112 compounds was designed. This library was selected for structural diversity (MolPrint2D fingerprint and Tanimoto coefficient <0.5), small drug-/lead-like size (MW 300–350), solubility (LogS > −4 and LogP < =3.5), flexibility (2–7 rotatable bonds), polarity (hydrogen bond donors + hydrogen bond acceptors + charged atoms = 3–10) and lack of reactive groups. The physicochemical properties were calculated by employing LigPrep, Epik, QikProp from Schrödinger Maestro and cxcalc form ChemAxon. To enable primary assay hits to be optimised by an ‘analogue-by-catalogue’ approach, we selected library compounds with at least 50 available drug-like analogues (Tanimoto coefficient > 0.55). A diversity search was conducted in the pool of screening compounds by Schrödinger Canvas (molPrint2D fingerprint and Tanimoto Coefficient = <0.5). The library was ordered ready for assaying as freeze-dried compounds in 384-well plates.

Substructure queries were constructed in MarvinSketch 17.3.27.0, 2017, ChemAxon (http://www.chemaxon.com) based on compound **2027** allowing for variations in different parts of the molecule. The actual analogue searches were performed in Instant JChem 17.2.27.0, 2017, ChemAxon (http://www.chemaxon.com) in a database of commercially available compounds combined from different vendors.

### Cell culturing and transient transfection

HEK293 cells were maintained in DMEM containing GlutaMAX-I, supplemented with 10% FBS and 1% penicillin-streptomycin and incubated at 37 °C and a humidity of 5% CO_2_. For expression of δ-containing GABA_Α_ receptors, a HEK293 Flp-In^ΤΜ^ cell line stably expressing the human GABA_Α_ δ-subunits (referred to as δ-HEK) was used and the γ-containing subtypes was expressed in a background HEK293 Flp-In^ΤΜ^ cell line stably expressing the G-protein coupled peptide receptor NPBWR2 (referred to as HEK background)^26^, both positively selected for using 200 μg/mL hygromycin B. α_1_β_2_γ_2_ receptors were stably expressed in a HEK293 cell line maintained as above but with no selection (gift from Marianne L. Jensen, Neurosearch).

To express recombinant GABA_Α_ receptors human α_1_, α_2_, α_3_, α_5_, α_6_, β_2_, γ_2_ (pcDNA3.1/Zeo) and α_4_, β_1_ (pUNIV)^26^ subunit cDNA were transiently transfected into either δ-HEK or HEK background cells. δ-HEK cells were transfected with α- and β-subunits in a 1:1 ration whereas HEK background cells were transfected with α-, β- and γ-subunits in a 1:1:2 ratio. Human BGT1 (pcDNA5/FRT)^43^ was transiently transfected into the δ-HEK cell line. δ-HEK cells transfected for whole-cell patch-clamp recordings were co-transfected with green fluorescent protein (GFP) as well as α- and β-subunits in a 0.5:1:1 ratio (0.8 μg + 1.6 μg + 1.6 μg in a 6 cm dish) in order to identify transfected cells. The transfection was carried out using Polyfect as transfection reagent according to the manufactures protocol expect for using half the recommended volume of Polyfect.

### FLIPR membrane potential (FMP) assay

The FMP assay was performed as previously described with a few modifications^26^. Briefly, HEK cells were transfected as described^26^ and 16–20 hours later plated (50,000 cells/well in DMEM medium) into clear-bottomed black 96-well plates coated with poly-D-lysine and the assay performed 20–28 hours later. The assay protocol including the FMP blue dye incorporation into membranes was exactly as described in Falk-Petersen *et al*.^26^. Ligand solutions were prepared in 4x in assay buffer (HBSS supplemented with 20 mM HEPES, pH 7.4, on the day of assay supplemented with 2 mM CaCl_2_ and 0.5 mM MgCl_2_, which for all compounds tested for antagonism contained a concentration of GABA corresponding to approximately GABA EC_80_ (given in Table 1). The ligand solutions were added to a ligand plates and incubated for 10–15 min in the FLEXstation3 plate reader (Molecular Devices, Crawley, UK) pre-heated to 37 °C. In a few cases a similarly operating machine, NOVOStar^ΤΜ^ (BMG LABTECH GmbH, Offenburg, Germany) was used instead, giving similar results. Data was obtained as changes in fluorescence units (∆RFU) by measuring emission at 560 nm caused by excitation at 530 nm. Unless otherwise mentioned in the text or figure legends experiments were performed in at least three independent experiments each using three technical replicates. Due to an observed edge effect for the FMP assay, outer wells were not used except for the screening. For screening results with GABA EC_80_ controls giving signals of less than 60 ∆RFU in the outer wells, the compounds were retested.

Data obtained in the FMP assay are given as relative changes in fluorescence units (ΔRFU) by subtracting the average of the baseline fluorescence signal from the peak fluorescence signal after compound addition. All raw traces were inspected manually and peak signals resulting from compound/buffer addition together with signal artefacts were omitted from the analysis.

The concentration-response curves obtained in the FMP assay for both agonists (determining EC_50_ values) and antagonists (determining IC_50_ values) were fitted using the four-parameter concentration-response model:

$${\rm{Response}}={\rm{bottom}}+\frac{{\rm{top}}-{\rm{bottom}}}{{1+10}^{{[({\rm{logEC}}}_{50}-{\rm{A}})\cdot {n}_{H}]}}$$

EC/IC_50_ values describe the concentration resulting in the half-maximal response (halfway between top and bottom), and n_Η_ is the Hill coefficient. In order to pool the data from the individual experiments for the Schild analysis, ΔRFU were normalized to the buffer level set as 0% response and average response of the highest tested concentration of GABA was set to 100% in order to compensate for variation in the assay window between experiments. The presence of **018** did not result in a decreased plateau of the curves, as the maximum response was similar to the GABA max (100 μM) controls. Gaddum Schild analysis was performed using GraphPad Prism version 8.2.1 (GraphPad Software Inc., San Diego, CA, USA). EC_50_/IC_50_ values obtained in the FMP assay are given as means ± SEM from at least three independent experiments with three technical replicates, as specified in the individual figure legends.

### Z′-factor determination

The Z′-factor for the FMP assay at α_4_β_1_δ receptors was calculated using the equation:

$${\rm{Z}}{\prime} =1-\frac{3{\sigma }_{c+}+3{\sigma }_{c-}}{|{\mu }_{c+}-{\mu }_{c-}|}$$

with σ_c-_ and σ_c+_ denoting the standard deviation of the negative and positive controls, respectively, and μ_c+_ and μ_c-_ the means of the positive and negative controls, respectively. GABA EC_80_ was the positive control and the buffer response the negative control.

### Whole-cell patch-clamp electrophysiology

δ-HEK cells transiently expressing human α_4_β_1_δ receptors were seeded in 35 mm Petri dishes the day before experiment and used for recordings in a similar was as previously reported^26^. On the day of experiment the dishes were transferred to the stage of an Axiovert 10 microscope (Zeiss, Germany), and the culture medium was exchanged for artificial balanced salt solution (ABBS) at room temperature (20−24 °C). The ABSS solution contained the following (in mM):  NaCl 140, KCl 3.5, Na_2_HPO_4_ 1.25, MgSO_4_ 2, CaCl_2_ 2, glucose 10, and HEPES 10; pH 7.35. The cells were viewed at 200× magnification, and cells containing green fluorescent protein were visualized with UV light from an HBO 50 lamp (Zeiss, Germany). The cells were approached with micropipettes of 1.2−3.3 MΩ resistance manufactured from 1.5 mm OD glass (World Precision Instruments, Sarasota, Florida) on a microelectrode puller, model PP-830 (Narishige, Tokyo, Japan). The intrapipette solution contained the following (in mM):  KCl 140, MgCl_2_ 1, CaCl_2_ 1, EGTA 10, MgATP 2, and HEPES 10; pH 7.3. Standard patch-clamp techniques in voltage clamp mode^44^ were used to record from cells in the whole-cell configuration using an EPC-9 amplifier (HEKA, Lambrecht, Germany). A clamping potential of −60 mV was used, and series resistance was 80% compensated. Whole-cell currents were recorded and subsequently analyzed using Pulse and PulseFit software (HEKA, Lambrecht, Germany).

ABSS solution containing the compounds were applied using two VC3–8xP pressurized application systems feeding into a sixteen-barreled perfusion pipette (ALA Scientific Instruments Inc., Farmingdale New York, USA) ending approximately 100 μm from the recorded cell. Between the compound applications, compound-free ABSS was applied from one of the barrels in order to quickly remove the compounds from the cell. For the concentrations-response dependent experiments, antagonist was pre-applied for 30 sec before 5 sec co-application with GABA EC_40_ (2.5 μM), with 1 min intervals between the applications to let the cells fully recover. For the kinetic studies GABA was first applied for 5 sec, followed by 5 sec application of antagonist and last 5–15 sec application of GABA, depending on when a plateau was reached. The cells were left to recover for 1 min before the next application.

For the antagonist concentration-response relationship, the equation:

$${\rm{I}}=\frac{{{\rm{I}}}_{{\rm{\max }}}}{1+{10}^{[({{\rm{logIC}}}_{50}-{\rm{A}})\cdot {{\rm{n}}}_{{\rm{H}}}]}}$$

was fitted to the experimental data, where I is the membrane current, A is the logarithm of the antagonist concentration, I_max_ is the maximum current that the reference agonist can induce, IC_50_ is the antagonist concentration inhibiting 50% of I_max_, and n_Η_ is the Hill coefficient. Data were described using means and 95% confidence intervals given en squared brackets.

Current relaxations were fitted to a single or biexponential equation using a Simplex optimization algorithm (PulseFit; HEKA, Lambrecht, Germany).

$${\rm{I}}({\rm{t}})={{\rm{I}}}_{0}+{{\rm{I}}}_{1}\exp \left(\frac{-{\rm{t}}}{{{\rm{\tau }}}_{1}}\right)+{{\rm{I}}}_{2}\exp \left(\frac{-{\rm{t}}}{{{\rm{\tau }}}_{2}}\right)$$

With I denoting the currents at a time t and τ_1_ and τ_2_ the time constants. All curve-fitting results were carefully inspected visually. In some cases, the current relaxations were fitted to both a mono- and a biexponential function and an *F*-test was applied to the residual sum of squares to determine the appropriateness of using a second component:

$$F=\frac{({{\rm{SS}}}_{{\rm{A}}}-{{\rm{SS}}}_{{\rm{B}}}){{\rm{df}}}_{{\rm{B}}}}{{{\rm{SS}}}_{{\rm{B}}}({{\rm{df}}}_{{\rm{A}}}-{{\rm{df}}}_{{\rm{B}}})}=\frac{({{\rm{RMS}}}_{{\rm{A}}}^{2}-{{\rm{RMS}}}_{{\rm{B}}}^{2}){{\rm{df}}}_{{\rm{B}}}}{{{\rm{RMS}}}_{{\rm{B}}}^{2}({{\rm{df}}}_{{\rm{A}}}-{{\rm{df}}}_{{\rm{B}}})}$$

where SS and RMS are the sum of squares and root mean square deviation between fit and data, respectively. A and B refer to the single and biexponential fit, respectively. df represents the degree of freedom for each fit. Time constants were given as medians followed by the 25–75% percentile in square brackets. All statistical analyses performed on time constants were performed by using non-parametric tests (Kruskal-Wallis ANOVA followed by Dunn’s multiple comparison), as the data do not follow a normal distribution.

All fractional amplitudes (%A) were determined from the second GABA pulse, for those concentrations of antagonist where a biexponential time course could be superimposed.

### Radioligand binding assay

The binding studies were performed using rat brain homogenate prepared from the cortex of adult Sprague-Dawley male rats (250–350 g) obtained from commercial breeders (Janvier Laboratories, Le Genest-Saint-Isle, France), housed and cared for according to the Danish legislation regulating animal experiments and the European Communities Council Directive (2010/63/EU). Homogenate preparation was performed as described earlier^45^. On the day of assay, membranes stored at −20 °C was quickly thawed followed by addition of 40 volumes of ice-cold binding buffer (50 mM TRIS-HCl buffer; pH 7.4) and homogenization using and UltraTurrax homogenizer before being centrifuged at 48,000 ×*g* for 10 minutes at 4 °C. This washing procedure was repeated 4 times before the final pellet was suspended in the binding buffer to final concentration of approximately 0.5 mg/mL protein.

The radioligand binding assay was performed using [^3^H]muscimol in a 96-well format. [^3^H]muscimol (5 nM) was incubated with 75 μg/well protein with or without test compound or control at 0 °C for 1 hour in a total volume of 200 μL binding buffer in triplicates. GABA (1 mM) was used to determine non-specific binding. After incubation, the binding reaction was terminated by rapid filtration through GF/C unifilters (PerkinElmer) with the use of a 96-well Packard FilterMate cell harvester (PerkinElmer), followed by 3 times washing in 250 μL ice-cold binding buffer. After drying at 65 °C, microscintillation fluid was added to the dried filters and the radioactivity bound to the filter was quantified in a Packard TopCounter microplate scintillation counter. The obtained binding data was converted into specific binding (% of control) for each data point and fitted to the one-site competition model by linear regression (GraphPad Prism, version 8.2.1). The fitted IC_50_ values were converted into K_i_ values by means of the Cheng-Prusoff equation^46^. Data are from three independent experiments, performed with three technical replicates and K_i_ values are given as pK_i_ ± SEM.

### Membrane permeability

Bidirectional permeability was tested for **018** and **2027** in the Madin-Darby canine kidney (MDCK) cell line expressing human multidrug resistance protein (MDR1, P-glycoprotein) (referred to as MDR1-MDCK cells) as described previously^47^. To calculate efflux ratio the permeability in the basal-to-apical direction was divided by the permeability in the apical-to-basal direction. The obtained data is from triplicate measurements.

### Data analysis and statistics

Detailed descriptions of data and statistics are stated in the respective method sections. Data and statistical analysis were performed using GraphPad Prism version 8.2.1 (GraphPad software Inc., San Diego, CA, USA).

## Supplementary information

Supplementary Information.

## Data Availability

The data generated and analysed during the present study is available from the corresponding author upon request.

## References

[1] Sieghart W, Sperk G (2002). Subunit composition, distribution and function of GABA_A_ receptor subtypes. Curr. Top. Med. Chem..

[2] Olsen RW, Sieghart W (2009). GABA_A_ receptors: subtypes provide diversity of function and pharmacology. Neuropharmacology.

[3] Farrant M, Nusser Z (2005). Variations on an inhibitory theme: phasic and tonic activation of GABA_A_ receptors. Nat. Rev. Neurosci..

[4] Pirker S, Schwarzer C, Wieselthaler A, Sieghart W, Sperk G (2000). GABA_A_ receptors: immunocytochemical distribution of 13 subunits in the adult rat brain. Neuroscience.

[5] Saxena NC, Macdonald RL (1994). Assembly of GABA_A_ receptor subunits: role of the δ subunit. J. Neurosci..

[6] Semyanov A, Walker MC, Kullmann DM, Silver RA (2004). Tonically active GABA_A_ receptors: modulating gain and maintaining the tone. Trends Neurosci..

[7] Caraiscos VB (2004). Tonic inhibition in mouse hippocampal CA1 pyramidal neurons is mediated by α5 subunit-containing γ-aminobutyric acid type A receptors. Proc. Natl. Acad. Sci. USA.

[8] Brickley SG, Mody I (2012). Extrasynaptic GABA_A_ receptors: their function in the CNS and implications for disease. Neuron.

[9] Lie MEK (2019). GAT3 selective substrate L-isoserine upregulates GAT3 expression and increases functional recovery after a focal ischemic stroke in mice. J. Cereb. Blood Flow Metab..

[10] Clarkson AN, Huang BS, MacIsaac SE, Mody I, Carmichael ST (2010). Reducing excessive GABA-mediated tonic inhibition promotes functional recovery after stroke. Nature.

[11] Mesbah-Oskui L, Orser BA, Horner RL (2014). Thalamic δ-subunit containing GABA_A_ receptors promote electrocortical signatures of deep non-REM sleep but do not mediate the effects of etomidate at the thalamus *in vivo*. J. Neurosci..

[12] Smith SS (2013). α4βδ GABA_A_ receptors and tonic inhibitory current during adolescence: effects on mood and synaptic plasticity. Front. Neural Circuits.

[13] Whissell PD (2013). Acutely increasing δGABA_A_ receptor activity impairs memory and inhibits synaptic plasticity in the hippocampus. Front. Neural Circuits.

[14] Collinson N (2002). Enhanced learning and memory and altered GABAergic synaptic transmission in mice lacking the α5 subunit of the GABA_A_ receptor. J. Neurosci..

[15] Storustovu SI, Ebert B (2006). Pharmacological characterization of agonists at δ-containing GABA_A_ receptors: functional selectivity for extrasynaptic receptors is dependent on the absence of γ_2_. J. Pharmacol. Exp. Ther..

[16] Wafford KA, Ebert B (2006). Gaboxadol - a new awakening in sleep. Curr. Opin. Pharmacol..

[17] Cogram P (2019). Gaboxadol normalizes behavioral abnormalities in a mouse model of fragile X syndrome. Front. Behav. Neurosci..

[18] Wafford KA (2009). Novel compounds selectively enhance δ subunit containing GABA_A_ receptors and increase tonic currents in thalamus. Neuropharmacology.

[19] Vardya I (2012). Positive modulation of δ-subunit containing GABA_A_ receptors in mouse neurons. Neuropharmacology.

[20] Karim N (2011). 3-Hydroxy-2’-methoxy-6-methylflavone: a potent anxiolytic with a unique selectivity profile at GABA_A_ receptor subtypes. Biochem. Pharmacol..

[21] Clarkson AN (2019). The flavonoid, 2’-methoxy-6-methylflavone, affords neuroprotection following focal cerebral ischaemia. J. Cereb. Blood Flow Metab..

[22] Hogenkamp DJ (2007). Enaminone amides as novel orally active GABA_A_ receptor modulators. J. Med. Chem..

[23] Johnstone TBC (2019). Positive allosteric modulators of nonbenzodiazepine γ-aminobutyric acid_A_ receptor subtypes for the treatment of chronic pain. Pain.

[24] Boddum K, Frølund B, Kristiansen U (2014). The GABA_A_ antagonist DPP-4-PIOL selectively antagonises tonic over phasic GABAergic currents in dentate gyrus granule cells. Neurochem. Res..

[25] Cope DW (2009). Enhanced tonic GABA_A_ inhibition in typical absence epilepsy. Nat. Med..

[26] Falk-Petersen CB (2017). Development of a robust mammalian cell-based assay for studying recombinant α_4_β_1/3_δ GABA_A_ receptor subtypes. Basic Clin. Pharmacol. Toxicol..

[27] Zhang C (1999). & Oldenburg. A simple statistical parameter for use in evaluation and validation of high throughput screening assays. J. Biomol. Screen..

[28] Glykys J (2007). A new naturally occurring GABA_A_ receptor subunit partnership with high sensitivity to ethanol. Nat. Neurosci..

[29] Maric D (1999). GABA_A_ receptor subunit composition and functional properties of Cl^-^ channels with differential sensitivity to zolpidem in embryonic rat hippocampal cells. J. Neurosci..

[30] Mortensen M, Patel B, Smart TG (2012). GABA potency at GABA_A_ receptors found in synaptic and extrasynaptic zones. Front. Cell. Neurosci..

[31] Ueno S, Bracamontes J, Zorumski C, Weiss DS, Steinbach JH (1997). Bicuculline and gabazine are allosteric inhibitors of channel opening of the GABA_A_ receptor. J. Neurosci..

[32] Mortensen M, Ebert B, Wafford K, Smart TG (2010). Distinct activities of GABA agonists at synaptic- and extrasynaptic-type GABA_A_ receptors. J. Physiol..

[33] Keramidas A, Harrison NL (2008). Agonist-dependent single channel current and gating in α_4_β_2_δ and α_1_β_2_γ_2S_ GABA_A_ receptors. J. Gen. Physiol..

[34] Heaulme M (1986). Biochemical characterization of the interaction of three pyridazinyl-GABA derivatives with the GABA_A_ receptor site. Brain Res..

[35] Xiao X, Zhu MX, Xu T (2013). Le. 2-Guanidine-4-methylquinazoline acts as a novel competitive antagonist of A type γ-aminobutyric acid receptors. Neuropharmacology.

[36] Ebert B (1997). Differences in agonist/antagonist binding affinity and receptor transduction using recombinant human γ-aminobutyric acid type A receptors. Mol. Pharmacol..

[37] Ebert B, Wafford KA, Whiting PJ, Krogsgaard-Larsen P, Kemp JA (1994). Molecular pharmacology of γ-aminobutyric acid type A receptor agonists and partial agonists in oocytes injected with different α, β, and γ receptor subunit combinations. Mol. Pharmacol..

[38] Chandra D (2010). Prototypic GABA_A_ receptor agonist muscimol acts preferentially through forebrain high-affinity binding sites. Neuropsychopharmacology.

[39] Quirk K, Whiting PJ, Ian Ragan C, McKernan RM (1995). Characterisation of δ-subunit containing GABA_A_ receptors from rat brain. Eur. J. Pharmacol. Mol. Pharmacol..

[40] Pálvölgyi A (2018). Loop F of the GABA_A_ receptor alpha subunit governs GABA potency. Neuropharmacology.

[41] Vestergaard HT, Cannillo C, Frølund B, Kristiansen U (2007). Differences in kinetics of structurally related competitive GABA_A_ receptor antagonists. Neuropharmacology.

[42] Barragan A, Weidner JM, Jin Z, Korpi ER, Birnir B (2015). GABAergic signalling in the immune system. Acta Physiol..

[43] Al-Khawaja A (2014). Pharmacological identification of a guanidine-containing β-alanine analogue with low micromolar potency and selectivity for the betaine/GABA transporter 1 (BGT1). Neurochem. Res..

[44] Hamill OP, Marty A, Neher E, Sakmann B, Sigworth FJ (1981). Improved patch-clamp techniques for high-resolution current recording from cells and cell-free membrane patches. Pflugers Arch..

[45] Ransom RW, Stec NL (1988). Cooperative modulation of [^3^H]MK‐801 binding to the N‐methyl‐D‐aspartate receptor‐ion channel complex by l‐glutamate, glycine, and polyamines. J. Neurochem..

[46] Yung-Chi C, Prusoff WH (1973). Relationship between the inhibition constant (K_I_) and the concentration of inhibitor which causes 50 per cent inhibition (I_50_) of an enzymatic reaction. Biochem. Pharmacol..

[47] Risgaard R (2013). Radiolabelling and PET brain imaging of the α_1_-adrenoceptor antagonist Lu AE43936. Nucl. Med. Biol..

[48] Krall J (2016). Discovery of α‐substituted imidazole‐4‐acetic acid analogues as a novel class of ρ_1_ γ‐aminobutyric acid type A receptor antagonists with effect on retinal vascular tone. ChemMedChem.

[49] L’Estrade ET (2019). Synthesis and pharmacological evaluation of [^11^C]4-methoxy-N-[2-(thiophen-2-yl)imidazo[1,2- a]pyridin-3-yl]benzamide as a brain penetrant PET ligand selective for the δ-subunit-containing γ-aminobutyric acid type A receptors. ACS Omega.

