# Supplementary material for: Discovery of a new class of orthosteric antagonists with nanomolar potency at extrasynaptic GABAA receptors
Source: Sci Rep. 2020 Jun 22;10:10078. doi: 10.1038/s41598-020-66821-0 (PMC7308271; doi:10.1038/s41598-020-66821-0)
Supplement: Supplementary file 1 — Supplementary Information. [file 41598_2020_66821_MOESM1_ESM.pdf]

Supplementary Information

Discovery of a new class of orthosteric antagonists with nanomolar potency at extrasynaptic $GABA_A$ receptors

Christina Birkedahl Falk-Petersen[1], Tsonko M. Tsonkov[1], Malene Sofie Nielsen[1], Kasper Harpsøe[1], Christoffer Bundgaard[2], Bente Frølund[1], Uffe Kristiansen[1], David E. Gloriam[1] and Petrine Wellendorph[1]*

[1]Department of Drug Design and Pharmacology, Faculty of Health and Medical Sciences, University of Copenhagen, Universitetsparken 2, 2100 Copenhagen Ø, Denmark. [2]Translational DMPK, H. Lundbeck A/S, Ottiliavej 9, 2500 Valby, Denmark. *Correspondence and requests for materials should be addressed to P.W. (email: pw@sund.ku.dk)

Supplementary Fig. S1

Validation of hits from the primary screening on $\alpha_4\beta_1\delta$ receptors in the FMP assay. Only **2027** was able to inhibit the GABA EC_{80} induced response in a concentration of 10 µM. The shown data is from a single experiment performed in triplicate, shown as means ± SD.

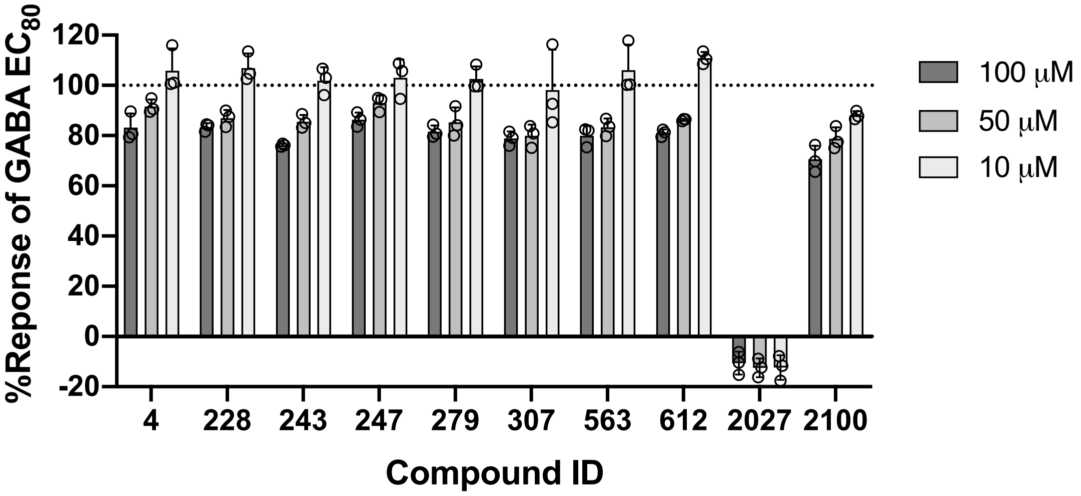

Supplementary Fig. S2

Structures and IDs of the 52 tested analogues of **2027**. Compounds were purchased from Enamine, except for compounds marked with * were purchased from Vitas-M and ** from ChemBridge.

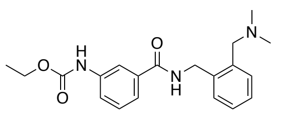

001
ID Z102680476

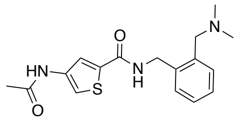

002
ID Z212333174

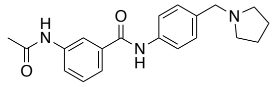

003
ID Z73418232

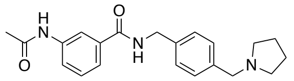

004
ID Z95955375

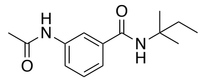

005
ID Z54128603

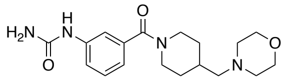

006
ID Z431707444

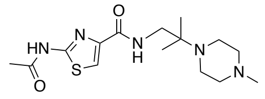

007
ID Z403738086

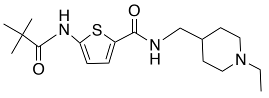

008
ID Z815037328

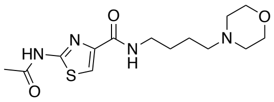

009
ID Z435166282

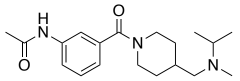

010
ID Z852202458

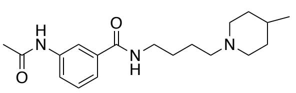

011
ID Z787433848

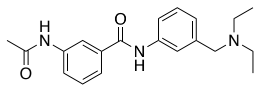

012
ID Z359400276

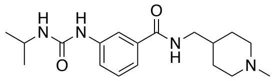

013
Z994125172

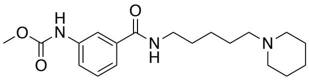

014
Z1173380151

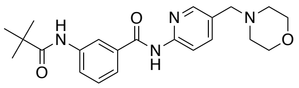

015
Z1530005336

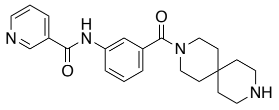

016
Z1840322786

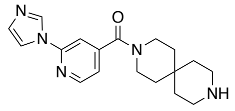

017
Z1840328432

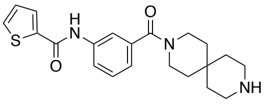

018
Z1839935473

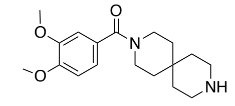

019
Z1839931153

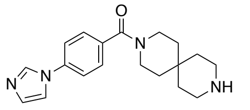

020
Z1840318505

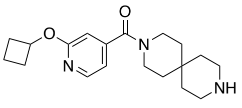

021
Z1840317511

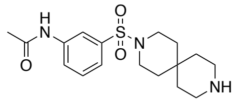

022
Z2040111683

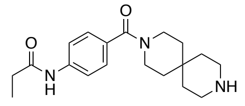

023
Z1839939872

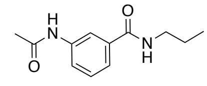

024
Z33031151

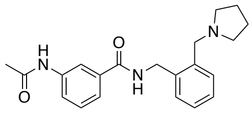

025

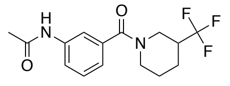

026

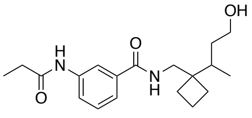

027

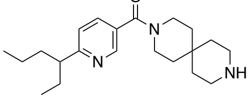

028

Z110039142 Z69129097 Z1594595161 Z1839943063

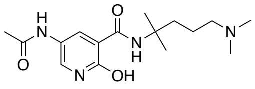

029
Z2690311908

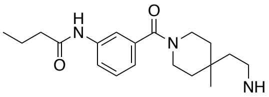

030
Z1839943489

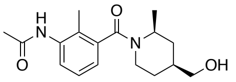

031
Z2203362952

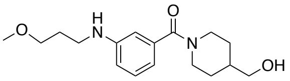

032
Z1270717092

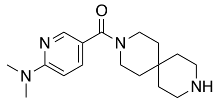

033
Z1839933645

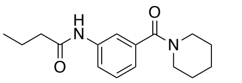

034
STL261341*

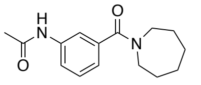

035
ID 7933531**

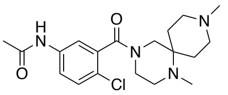

036
ID 11690210**

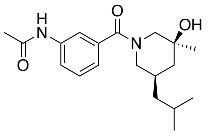

037
ID 21874358**

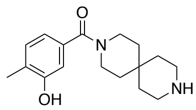

038
ID 25282273**

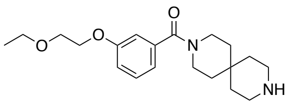

039
ID 25386588**

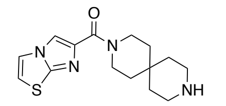

040
ID 41457308**

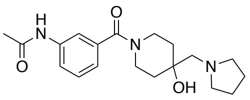

041
ID 55948614**

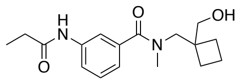

042
ID 56799745**

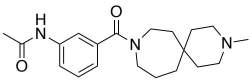

043
ID 62708052**

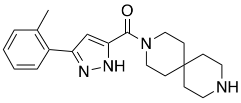

044
ID 63219423**

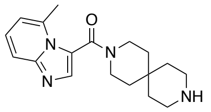

045
ID 67473017**

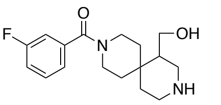

046
ID 69138959**

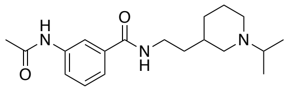

047
ID 70226034**

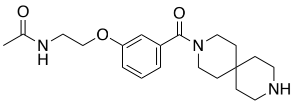

048
ID 73503019**

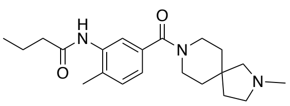

049
ID 77120366**

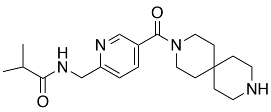

050
ID 77296929**

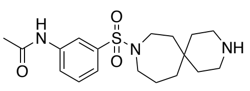

051
ID 79549842**

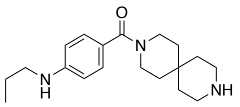

052
ID 89524344**

Supplementary Fig. S3

Results of the testing of the 52 analogues of **2027** at $\alpha_4\beta_1\delta$ receptors in the FMP assay. Compounds were tested in concentrations of 0.5 μM and 5 μM in duplicates in a single experiment. Gabazine (GBZ) in a concentration of 5 μM was included as a positive control. Compound ID refer to supplementary Fig. S2. The shown data is given as means ± SD from a single experiment.

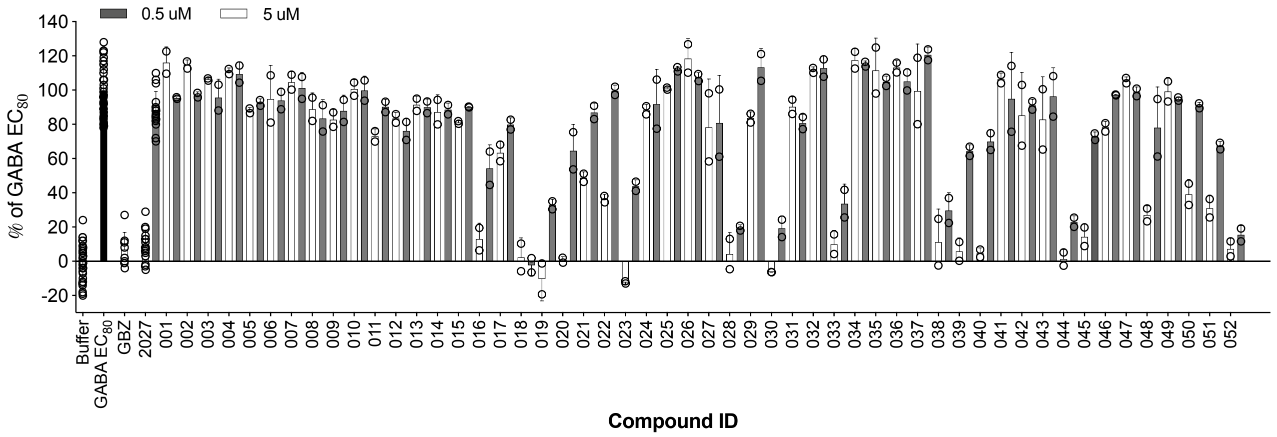

Supplementary Fig. S4

Compound **018** shows slow dissociation kinetics independent of the GABA concentration. (**A**) Time constants, τ, for currents induced by 100 µM GABA with or without preincubation of **018**. The constants were determined by fitting to a monoexponential function for no **018** preapplication and 1 nM **018** preapplication and to a biexponential function for the remaining cells except for 3 cells applied 3 nM **018** and 1 cell applied 30 nM **018** which could be fitted to both a mono- and biexponential function. F-test showed that these cells were best fitted to the biexponential function (F-test, $P<0.05$). Statistical analysis was performed using Kruskal-Wallis ANOVA followed by Dunn's multiple comparison. τ_1 was compared to 10 nM **018** (100 µM GABA alone). Data are shown as medians with interquartile range (25% and 75%). (**B**) Concentration-dependent decrease in $\%A_1$ for 100 µM GABA, giving a functional K_B of 6.9 nM [5.99:7.91], n=5-6. Data are given as means ± SEM. Further details are given in Table S1.

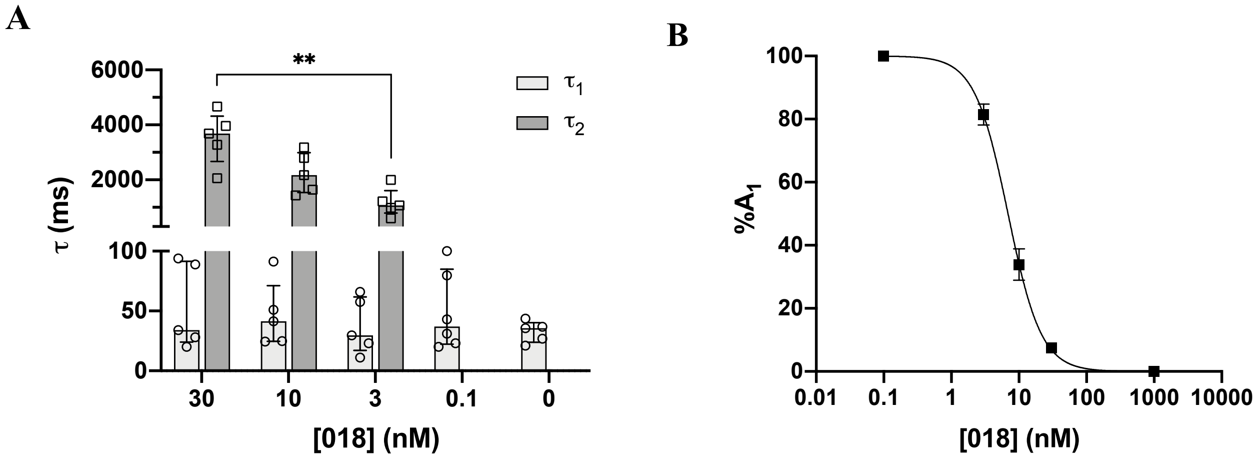

Supplementary Fig. S5

Compound **2027** does not quench the fluorescent signal induced by BGT1-mediated GABA uptake in the FMP assay (performed essentially as previously described in methods section and based on Al-Khawaja et al. [43]). As shown, the fluorescent signal induced by uptake of 110 μM GABA by human BGT1 transiently expressed in δ-HEK cells was not decreased/quenched by **2027** in any of the tested concentrations. Data are shown as means ± SD from a representative experiment performed in triplicate (n=2).

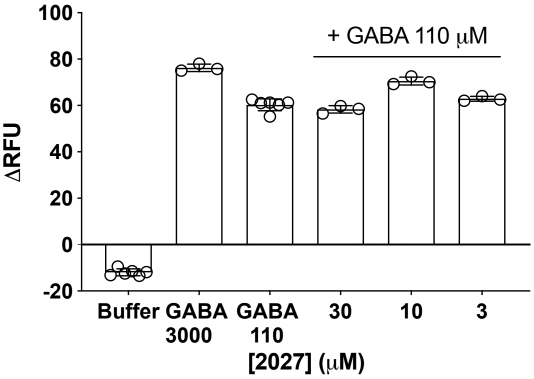

Supplementary Table S1

Summary of time constant for 100 µM GABA with or without preapplication of **018** and the contribution from the fast current amplitude to the total amplitude (%A_1).

**018** (nM)	τ_1 (ms)		τ_2 (ms)		**%A_1**	**n**
0	35.0	[24.0;40.0][a]	-		-	5
0.1	37.1	[22.3;84.9][a]	-		-	6
3	29.6	[22.4;61.8]	1070	[793;1607]	81.5	5
10	41.4	[24.6;71.2]	2170	[1535;2990]	33.9	5
30	34.0	[24.0;91.5]	3685	[2670;4315]	7.5	5

If not stated, current traces were fitted to a biexponential function. 3 cells applied with 3 nM **018** and 1 cell applied with 30 nM **018** could be fitted to both a mono- and biexponential function. F-test showed that they were best fitted to the biexponential function (F-test, $P<0.05$). Data are given as medians followed by 25-75% quartiles in squared brackets with *n* denoting the number of tested cells. Comparison of the determined time constant are shown and described in supplementary Fig. S4. [a] Current traces could only be fitted to a mono-exponential function.

Supplementary Table S2

The permeability study of **018** and **2027** shows high degree of recovery both before and after cell lysis indicating that sticking to the cells was no issue.

Compound	Mean recovery %		Mean total recovery %	
	Apical to basal	Basal to apical	Apical to basal	Basal to apical
**018**	97.93	101.17	99.59	101.17
**2027**	87.93	104.62	89.44	104.62
